# Some Mistaken Impressions Regarding Troubles of the Genito-Urinary Organs1Read before the Physicians’ Club, September 12, 1900, and at the thirty-third annual meeting of the Medical Association of Central New York, held at Rochester, N. Y., October 16, 1900.

**Published:** 1901-03

**Authors:** J. Henry Dowd

**Affiliations:** Buffalo, N. Y.


### SOME MISTAKEN IMPRESSIONS REGARDING TROUBLES OF THE GENITO-URINARY ORGANS.[1]

By J. HENRY DOWD, M. D., Buffalo, N. Y.

IT IS only during recent years that much attention has been given to inflammatory diseases of the genito-urinary organs, and especially when the same has been caused by venereal infection.

For this reason many medical men, I might say the great majority, are working on theories at least one hundred years old and these having been carried down by medical writers until say from five to eight years ago. It must be surmised from the above statement, that graduates previous to that time are now working under these old theories, unless they are, giving or have given most careful consideration to diseases of these organs since their college lectures. Even books of today are slightly hazy on certain conditions involving the genital and urinary organs. For instance, in the latest publication the opacity of urine in chronic urethral trouble is spoken of as being due to mucus and a slight amount of pus, when in reality there is very little of the former, the turbidity in nearly, if not every case being due to pus; the same being positively demonstratable if the microscope has been used. Many of these fallacious opinions have been so ground, into the laity, that it is a common occurrence to hear a patient say that he had an inflammation of the bladder with his gonorrhea, where by careful questioning the fact is brought out that he was suffering only from an involvement of the posterior urethra. The diagnostician in every case was a doctor, one who has been taught that frequency of urination, with pus in the second glass, meant cystitis.

*Cystitis Complicating Gonorrhea.*—Although every case of acute gonorrhea in the male is accompanied by frequency of urination, still involvement of the bladder during this disease is of the rarest occurrence. To understand this thoroughly one should be intimately acquainted with the physiology of urination. In perfect health and under normal conditions, urine enters the bladder at the average rate of about two ounces every hour. When the viscus becomes filled to an extent sufficient to overcome the resistance of the internal sphincter, this muscle relaxes, allowing the fluid to trickle into the posterior urethra, which at this time becomes a part of the urinary

1. Read before the Physicians' Club, September 12, 1900, and at the thirty-third annual meeting of the Medical Association of Central New York, held at Rochester, N. Y., October 16, 1900.

bladder, the neck. If there is inflammation in this portion of the canal and it is acute, as soon as the first drop of urine strikes the inflamed membrane, there is immediate desire to urinate, and but little, if any pus will be found in the second glass, for the reason that time did not permit of the pent up product of inflammation to gravitate backward and mix with the urine in the bladder.

On the other hand, supposing the membrane has been in a state of inflammation sufficiently long to withstand a certain amount of irritation, as soon as the internal sphincter gives away the urine enters, but the desire to urinate does not take place, rather the pus and other débris, intermingles with the incoming fluid, is carried into the bladder by the constant agitation which is going on, and when the desire is felt, if the urine is caught in ten divided parts, each and every one will contain pus, yet no involvement of the bladder is present.

As there is no doubt that the posterior urethra is involved in every case of gonorrhea, it must be very evident to all that the germs find their way, sooner or later into the bladder. Various theories have been advanced as to the cause of gonorrheal cystitis, but I shall only offer an explanation as to why this viscus is so rarely affected. Although the epithelium lining the bladder appears similar to that of others parts of the anatomy, it seems to have a resisting power that is found in only one other structure, the vagina. Above the bladder is found two organs of the utmost importance to life, and it seems that nature has taken into consideration the frequency of urethral inflammation and has placed this barrier, the bladder, between this canal and the aforementioned organs. The female bladder also withstands the attack of inflammation well, yet it is more prone to inflammation than that of the male. It can be briefly stated that cystitis is rarely an accompaniment of acute, or even chronic gonorrhea, when there is no other complicating factor present.

*Vaginitis.*— In the female it is known that there exists a direct route from the outside world to the peritoneal cavity and it seems that nature has interposed a fortress between for the protection of that delicate portion of her anatomy. We find this tube, the vagina, to be lined with epithelium which seems to have a resisting power against germ invasion and at the same time, and in contradistinction to the bladder of the male, the habitat of germs which, of no serious consequence to their possessor, has the power of destroying any foreign germ that may seek an entrance.

It may be inferred that the question will be asked, "how can pelvic peritonitis be accounted for, or, if infection does take place at the cervix, do not the germs gravitate downward in the secretions and in this way infect the vagina?" Suffice it to say, the resisting epithelium, together with the bacteria inhabiting the part, offer an obstruction so great, that even the gonococci in their most virulent form, except in extreme conditions of general systemic debility, fail to find a foothold in this portion of the human anatomy. Excluding traumatism, in the adult inflammation of the vagina is very, very rare. From careful examinations the writer has seen but two cases in five years; both were due to gonorrhea and both were syphilitics.

*Albumin and casts as indicative of nephritis.*—The old idea that when urine is boiled, becomes turbid, and this turbidity is not cleared away by the addition of nitric acid, that albumin is present, and that this is an indication of nephritis, is an exploded theory. It is not an uncommon thing to find the urine loaded with albumin during an attack of urethral inflammation where the most careful examination shows positively that no kidney lesion exists. It should be remembered that whenever serum exudes from the bloodvessels, albumin will be found in the urine, and the leaking of this substance (serum) is quite frequently found during an attack of gonorrhea or other inflammation involving the urethra or the bladder. Wherever there is a great amount of pus, the finest tests will always show a slight amount of albumin, but the kidneys must not be accused of producing the same, unless these organs are positively known to be involved. Albumin in discoverable quantities is not a constant accompaniment of Bright's disease; on the other hand, it is seldom found in the chronic forms, such as the interstitial. The finding of casts has generally been thought to be a most positive indication of nephritis, but such is not the fact in all cases. It is the most common occurrence to find a hyaline cast, especially if the centrifugal machine is used, and even epithelial casts are occasionally seen. If one will take the trouble to examine his urine carefully after a meal, rich in substances known to cause kidney irritation, also the free use of alcohol, many casts will be seen, hyaline, epithelial, and even granular; still, the kidneys are only suffering from a circulatory change. Of course, it must be inferred that the epithelium on the casts is in a healthy condition, for if it shows degeneration, it is indicative of a lesion.

*Syphilis.*—Many important points regarding this disease are open to doubt in the minds of not only the profession, but the laity. Syphilis must be included under the curable maladies, excepting where it assumes a malignancy from the outset, or is allowed to reach the tertiary stage. No attempt must be made to destroy a syphilitic chancre, as it very rarely accomplishes anything; but, on the other hand, causes a destruction of tissue which leaves a permanent scar. Tertiary symptoms may develop at any time, even as early as the sixth week after infection. The hot springs of Arkansas have no curative powers, they are only of benefit in cases of great debility, where the water acts as a tonic to the system. A positive prognosis must never be given in case of chancroid; there may be mixed infection.

*Stricture.*—Twisting or screw-like formation of the urine as it merges from the meatus is in no way indicative of urethral stricture. Herpes, in at least 95 per cent. of all cases are nature's warning that trouble exists in the canal, and this trouble is generally stricture. A sound will not diagnosticate a stricture and locate it, unless it is a very small one and a large instrument is used. Where chronic urethral inflammation complicates a stricture, never attempt dilatation until the urine, by its clearness, shows that the general inflammation has almost, if not entirely subsided. The urethra when to be operated upon, or manipulated, needs the same antiseptic precautions that any other part of the anatomy would receive. Internal medication is of little or no value in acute, and except in a very limited number of cases, is of no value whatever in chronic inflammation of the urinary tract, especially below the kidneys.

*Syphilis vs. gonorrhea.*—The common opinions held by the laity, that gonorrhea is no worse than a bad cold, and syphilis is never curable, and is transmitted to their children's children, is fast losing ground, and well it should. The statement can be made without much fear of contradiction, that of the two diseases syphilis is much the preferable, if we must choose between them. It is rare that death or even much deformity can be traced to syphilis, provided the disease is treated according to the methods in vogue today; whereas on the other hand, no one can foretell the ultimate result which may follow an infection from the gonococci. It is a very broad statement, but could gonorrhea be eliminated from the category of diseases affecting humanity, fully 1c per cent. of the suffering that the human being is subjected to would be a thing of the past. It should be remembered that gonorrhea kills more people than syphilis, although the latter takes years of protracted treatment for a cure.

288 FRANKLIN STREET.

